# Post-Stroke Psychiatric and Cognitive Symptoms in West Asia, South Asia and Africa: A Systematic Review and Meta-Analysis

**DOI:** 10.3390/jcm10163655

**Published:** 2021-08-18

**Authors:** Sangeetha Mahadevan, Moon Fai Chan, Marzieh Moghadas, Maithili Shetty, David T. Burke, Khalid Al-Rasadi, Samir Al-Adawi

**Affiliations:** 1 Department of Behavioural Medicine, College of Medicine and Health Sciences, Sultan Qaboos University, Muscat 123, Oman; sm5520@nyu.edu (S.M.); marziemoqadass@gmail.com (M.M.); maithili.shetty97@gmail.com (M.S.); 2 Department of Family Medicine & Public Health, College of Medicine & Health Sciences, Sultan Qaboos University, Muscat 123, Oman; moonf@squ.edu.om; 3 Department of Rehabilitation Medicine in the Emory University School of Medicine, 1441 Clifton Road N.E., Atlanta, GA 30322, USA; dburke2@emory.edu; 4 Department of Biochemistry, College of Medicine and Health Sciences, Sultan Qaboos University, Muscat 123, Oman; k.alrasadi@gmail.com

**Keywords:** post-stroke, depression, anxiety, cognitive impairment, prevalence, meta-analysis, systematic review, West Asia, South Asia, Africa

## Abstract

Recent research has shown that the prevalence of stroke incidents and the number of survivors in developing countries surpass those from developed countries. This study aimed to enumerate the prevalence of post-stroke psychiatric and cognitive symptoms among stroke survivors from West and South Asia and Africa through a systematic review and meta-analysis. Data from each country was systematically acquired from five major databases (PsycINFO, Web of Science, Scopus, PubMed/Medline, and Google Scholar (for any missing articles and grey literature)). Meta-analytic techniques were then used to estimate the prevalence of various post-stoke psychiatric and cognitive symptoms. A total of 36 articles were accrued from 11 countries, of which 25 were evaluated as part of the meta-analysis. The pooled prevalence of post-stroke depression as per the Hospital Anxiety and Depression Scale (HADS), Hamilton Depression Rating Scale, Patient Health Questionnaire, Schedules for Clinical Assessment in Neuropsychiatry (SCAN), Geriatric Depression Scale, and the Montgomery–Asberg Depression Rating Scale ranged from 28.00 to 50.24%. Pooled prevalence of post-stroke anxiety based on the HADS and SCAN was 44.19% and 10.96%, respectively. The pooled prevalence of post-stroke cognitive impairment as per the Mini-Mental Status Examination was 16.76%. This present review has suggested that both psychiatric and cognitive symptoms are common among stroke survivors. Concerted efforts are needed to institute robust studies using culturally sensitive measures to contemplate mechanisms that address the unmet needs of this vulnerable population.

## 1. Introduction

Recently, in the part of the world where approximately 80% of the global population resides—sometimes labeled as the ‘Global South’, ‘emerging economies’, ‘low- and middle-income countries’ or simply ‘developing’ or ‘non-Western countries’—there has been a surge of health problems resonating with the metaphor of a ‘*double*-*edged sword*’. On one hand, while environment-related and infectious diseases continue to pose challenges, these developing countries are concurrently witnessing a spike in non-communicable diseases including neurological events that lead to the intransigent and debilitating sequelae of focal (or global) disturbance in cerebral function. In this regard, stroke has been widely established to compromise the integrity of emotional and cognitive functioning, often going on to disrupt an individual’s premorbid self-directedness, quality of life, and meaningful existence. Stroke has also been recorded as being the second leading cause of death and dependency worldwide [1,2] and accounts for almost 5% of all disability-adjusted life-years and 10% of all deaths worldwide [3].

Research from many industrialized countries of North America, Western Europe and the pocket of countries in the Pacific Rim have shown that the most common risk factors contributing to the stroke epidemic include transient ischemic attacks, high blood pressure, tobacco smoking, end-stage kidney disease, atrial fibrillation disease, obesity, hyperlipidemia, and diabetes mellitus [4]. However, a rise in awareness campaigns, resource mobilization and the subsequent availability of emergency services, critical care, and rehabilitation services have contributed to the changing trends in these countries [5]. Such accessibility has also been shown to help pre-empt disability and dependency and the occurrence of death. In contrast, despite these debilitating health conditions being rife in many developing countries, with prevalence rates overtaking the West in some cases, the availability of the aforementioned resources is largely rudimentary [6]. Some populations, for example, those in South Asia, have been shown to have a high risk of stroke and account for almost 40% of the adverse stroke sequelae [7].

As a result, stroke survivors in developing countries have been documented to be marked with intransigent and debilitating disabilities [8]. To combat these dire circumstances, the World Health Organization (WHO) launched ‘Rehabilitation 2030′, which aspires to “scale up rehabilitation so that countries can be prepared to address the evolving needs of populations up to 2030” (p. 12) [9]. Owing to the suboptimal emergency services, critical care and existing rehabilitation services for individuals sustaining strokes in a majority of developing countries, stroke survivors have shown high mortality as well as disability and dependency rates [10]. Cerebral hypoperfusion among stroke survivors (whether triggered by ischemic or hemorrhagic events, coupled with arrhythmias, myocardial infarction, pulmonary embolism, pericardial effusion, or bleeding) leads to structural and functional changes in the brain resulting from damage. Around 85% of strokes are classified as ischemic and 12% as hemorrhagic [11]. Approximately 75% of stroke survivors experience physical disability, emotional symptoms and cognitive symptoms, or a combination of these. According to the ‘GBD 2016 Lifetime Risk of Stroke Collaborators’, over 80% of disability-adjusted life-years associated with stroke occur in low- and middle-income countries [12].

In the past decades, an increasing number of impressionistic and anecdotal reports along with a significant number of observational studies have reported the prevalence rates and types of emotional and cognitive symptoms persistent among stroke survivors in developing countries. In this regard, such studies must be applied in a wider context so that evidence-based quantification, prevention, and mitigation can be contemplated upon. To date, while there have been narrative reviews reported from developing countries [3,13], there are no critical appraisals such as systematic reviews or metanalyses on psychiatric and cognitive symptoms among stroke survivors.

While there is an abundance of research on cognitive and psychiatric symptoms secondary to stroke from many industrialized countries [14,15,16], little has been forthcoming from countries of the ‘global south’, despite showing a high prevalence of stroke. The established high variability in the incidence of post-stroke cognitive and psychiatric symptoms found across varied populations might imply that the role played by various idiosyncratic socio-cultural or ecological factors in determining the types and expression of these post-stroke sequelae is substantial [17,18]. Thus, complete reliance on the data from industrialized countries might not be viable when ameliorating the situation in developing countries. Additionally, a critical appraisal of the prevailing secondary post-stroke conditions in developing countries has the potential to help standardize the taxonomy relevant to a screening approach which, in turn, could aid in the efforts towards intervention, remedy, and rehabilitation. Furthermore, these taxonomic assumptions could be used for the allocation of resources, the making of administrative decisions, professional communication, diagnostic formulations, research, epidemiology, and public policy.

### Aims

This study aimed to calculate the prevalence of psychiatric and cognitive symptoms among stroke survivors from West and South Asia and Africa through a systematic review and meta-analysis.

## 2. Methods

The following systematic review was conducted via a properly established protocol, using the Preferred Reporting Items for Systematic Reviews and Meta-Analyses (PRISMA) guidelines [19] and included published articles up to the present year (2021). The extraction of articles began with the use of search terms across different levels delimited using the term “AND”. Level 1 (for stroke) included search terms such as “stroke” or “cerebrovascular disorders”, or “cerebrovascular accident”, or “Ischemic Stroke”, or “Intracerebral Hemorrhage”. Level 2 (for the mental disorder) included the following search terms: “Mental disorder(s)” or “Psychiatric Disorder(s)” or “Mental Illness(es)” or “adjustment disorders” or “neurotic disorders” or “cognitive impairment(s)” or “worry” or “fear” or other specific individual mental disorders such as “Depression”, “Anxiety”, “Eating disorder(s)”, “PTSD”, “dementia”, “cognitive decline”, or “executive (dys)function”, while the final level included the individual country names (GCC: Bahrain, Kuwait, Oman, Qatar, Saudi Arabia, and the United Arab Emirates. Western Asia: Iraq, Israel, Jordan, Lebanon, Palestine, Syria, Iran and Pakistan, Afghanistan, Bahrain, Kuwait, Oman, Qatar, Saudi Arabia, United Arab Emirates, and Yemen. South Asia: Bangladesh, Bhutan, India, Pakistan, Nepal, Sri Lanka, Afghanistan, and the Maldives. Africa: Algeria, Angola, Benin, Botswana, Burkina Faso, Burundi, Cabo Verde, Cameroon, Central African Republic (CAR), Chad, Comoros, Democratic Republic of Congo, Republic of Congo, Cote d’Ivoire, Djibouti, Egypt, Equatorial Guinea, Eritrea, Eswatini (formerly Swaziland), Ethiopia, Gabon, Gambia, Ghana, Guinea, Guinea-Bissau, Kenya, Lesotho, Liberia, Libya, Madagascar, Malawi, Mali, Mauritania, Mauritius, Morocco, Mozambique, Namibia, Niger, Nigeria, Rwanda, Sao Tome and Principe, Senegal, Seychelles, Sierra Leone, Somalia, South Africa, South Sudan, Sudan, Tanzania, Togo, Tunisia, Uganda, Zambia, Zimbabwe). The accrued articles were screened further to identify whether or not they met the required eligibility criteria.

### 2.1. Data Retrieval Strategies

The process of identifying articles based on the inclusion criteria began with a complete screening of the major databases: PsycINFO, Web of Science, Scopus, and PubMed/Medline. A final search of up to 14 pages on Google Scholar was also done to ensure the inclusion of grey literature and any remaining articles that may have been missed out. This chosen strategy did not include a search based on any specific timestamp, implying that all articles published before 6 May 2021 were included in the search.

**Figure 1:**
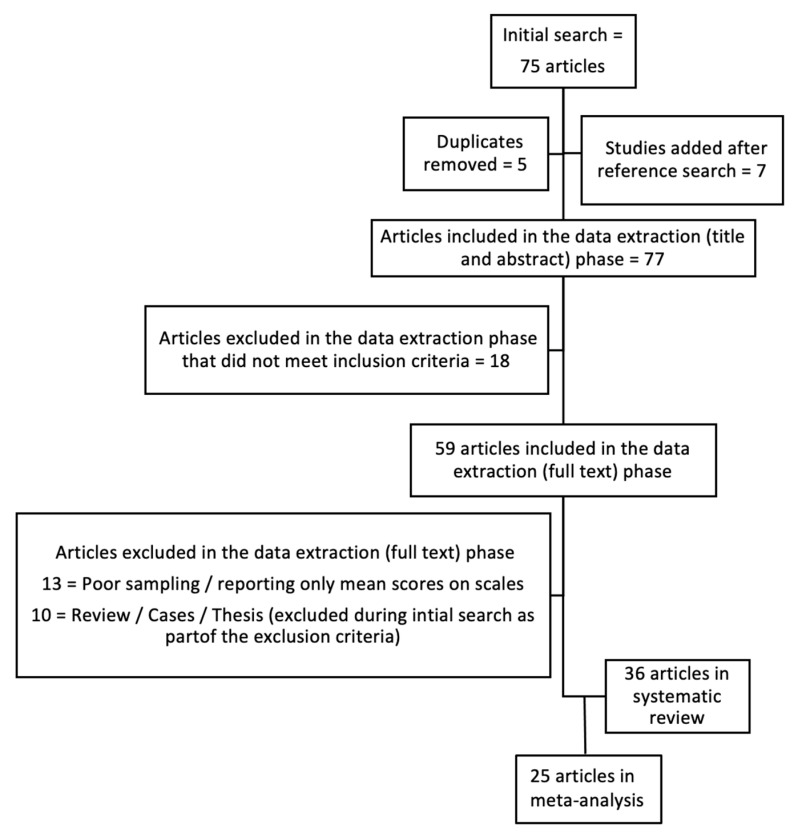
The PRISMA flow diagram describing the systematic review process.

As shown in Figure 1, the current search yielded a total of 75 usable articles. Following the exclusion of duplicates and inaccessible and irrelevant articles by three independent authors (MS, MM, and SM), there were a total of 70 articles. Complete articles were downloaded when the title and abstract of the article met the inclusion criteria. The reference lists of all included articles were then hand-searched for any articles that may have been missed out during the database search process to yield a final total of 59 articles for the full review for quality using the Joanna Briggs Institute (JBI) guidelines for the appraisal of scientific research articles [20]. The third and fourth authors were consulted when a disagreement arose between the three reviewers. This occurred for 2 of the 59 articles reviewed. Reviews using the JBI guidelines yielded a total of 36 articles that earned a score equal to or above the 75% cut-off point set for inclusion in this systematic review, of which 25 were finally included in the meta-analysis.

### 2.2. Inclusion and Exclusion Criteria

All studies included in this meta-analysis (1) were cross-sectional/cohort/case-control studies examining the prevalence of post-stroke secondary conditions in the general population; (2) used validated assessment tools; (3) had proper numerical information about prevalence and sample size; (4) were written in English. Articles were excluded if they were reviews, case reports, duplicated studies, non-human studies, or studies that did not include the examination of the prevalence of secondary post-stroke conditions as one of its aims. Additionally, studies were excluded if they scored below the 75% cut-off point on the evaluations while the JBI guidelines were being used. Articles were also excluded if they did not provide a proper prevalence measure of any of the secondary mental disorders as classified by the DSM-or ICD [21,22]. While the DSM, ICD, and the National Institute of Neurological and Communicative Disorders and Stroke and the AD and Related Disorders Association (NINCDS-ADRDA) [23] have defined what constitutes cognitive impairment, their criteria for cognitive impairment are quite diverse. There is also a tendency in the existing literature to often label symptoms of cognitive impairment as dementia. For the present purpose, cognitive impairment refers to the diminution of neuropsychological status that owes its development to its onset after stroke. Cognitive impairment has various derivatives including impaired attention and concentration, learning, and remembering, language, visuospatial ability, executive function and related neuropsychological domains [24]. Most of the studies reporting results on post-stroke secondary conditions have relied on instruments which, according to Harvey [25], gauge a “composite score across multiple ability areas to provide an overall index of how well a person functions cognitively at the current time” (p. 92). The studies that fulfilled the present inclusion criteria were those that employed composite scores across multiple cognitive domains. 

### 2.3. Data Extraction

Three authors (MM, MS, and SM) independently extracted relevant information from identified studies, including the name of the first author, the year of publication, the country in which the study was conducted, the year(s) in which the study was conducted, sampling methods, the mean, median, and the standard deviation of the age of participants, along with the age-range, the sample characteristic (university student, patient, etc.), total sample size, the gender distribution of the sample, the assessment tools, tool reliability, disorder screened, the total number of diagnosed cases, associated factors, and the amount of time elapsed between the stroke incident and the administration of the neuropsychological test (post-stroke duration).

### 2.4. Evaluation of the Quality of Reports on the Studies

Independent rating of the title, abstract, methods, results, discussion, and other parts of each included study according to the standard items listed in the Joanna Briggs Institute guidelines was carried out by the three individual reviewers [20]. The range of the total score was zero to eight, with each item accounting for one point (Table 1). The inter-rater reliability of the three authors for this quality measure was strong, with an intraclass correlation coefficient (ICC) of 0.8.

### 2.5. Statistical Analysis

Acquired data were analyzed using the MedCalc 12 statistical software. In this review, three main secondary conditions (depression, anxiety, and cognitive impairment) were identified for the PS patients. Some of the mental disorder outcomes were assessed by different tools. In the meta-analysis, the estimated pooled prevalence for each disorder outcome was calculated by using different assessment tools [51]. I^2^ and Q statistics were used to assess heterogeneity across articles [52]. For the heterogeneity test, If I^2^ statistic was greater than 50% and the Q statistic was < 0.1, a random-effects model was used to interpret the results; otherwise, we used the fixed-effects model [52,53].

## 3. Results

### 3.1. Study Characteristics

Although the initial search included 83 countries, the 11 countries that were finally included in this study were: Saudi Arabia, Qatar, Jordan, Iran, Egypt, Nigeria, Senegal, Uganda, India, Sri Lanka, and Bangladesh, with a total of 36 studies (Table 2). The highest number of studies were from Nigeria and India, accounting for 12 and 11 studies, respectively, followed by Egypt with five studies. The remaining countries of Saudi Arabia, Qatar, Jordan, Iran, Senegal, Uganda, Sri Lanka, and Bangladesh yielded one study each. The reported secondary conditions include post-stroke depression (PSD) (29 studies), post-stroke anxiety (PSA) (six studies), and post-stroke cognitive impairments (PSCI) (four studies).

### 3.2. Prevalence of Post-Stroke Depression (PSD) Assessed by the Hospital Anxiety and Depression Scale (HADS)

**Figure 2:**
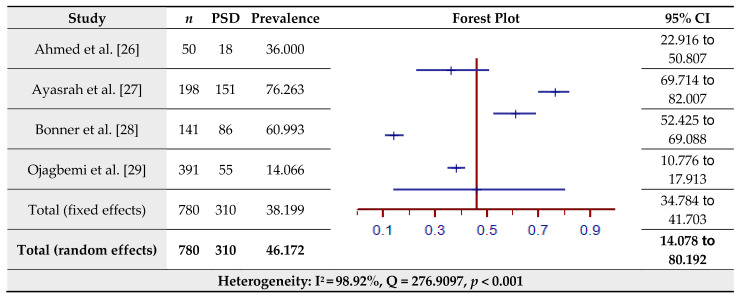
Prevalence estimates of Post-Stroke Depression (PSD) assessed by the Hospital Anxiety and Depression Scale (HADS).

The estimated prevalence of PSD assessed by the HADS for four studies is shown in Figure 2. The pooled prevalence of PSD in the total sample of 780 was 46.17% (95% CI = 14.08–80.19%) based on the random-effects model (I^2^ = 98.92%, Q = 276.91, *p* < 0.001).

### 3.3. Prevalence of Post-Stroke Depression (PSD) Assessed by the Hamilton Depression Rating Scale (HDRS)

**Figure 3:**
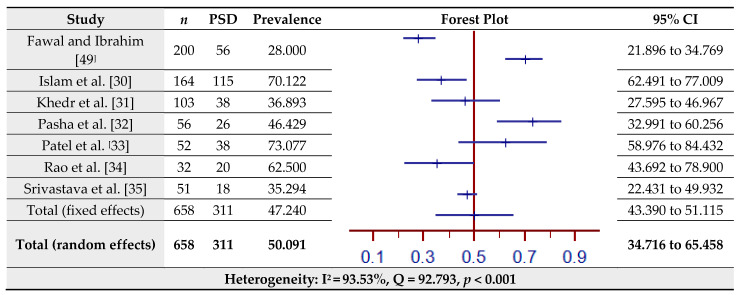
Prevalence estimates of Post-Stroke Depression (PSD) assessed by the Hamilton Depression Rating Scale (HDRS).

The estimated prevalence of PSD assessed by the HDRS for seven studies is shown in Figure 3. The pooled prevalence of PSD in the total sample of 658 was 50.09% (95% CI = 34.72–29.65.46%) based on the random-effects model (I^2^ = 93.53%, Q = 92.79, *p* < 0.001).

### 3.4. Prevalence of Post-Stroke Depression (PSD) Assessed by the Patient Health Questionnaire (PHQ-9)

**Figure 4:**
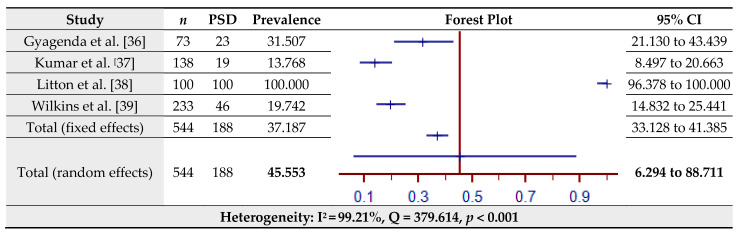
Prevalence estimates of Post-Stroke Depression (PSD) assessed by the Patient Health Questionnaire (PHQ-9).

**Figure 5:**
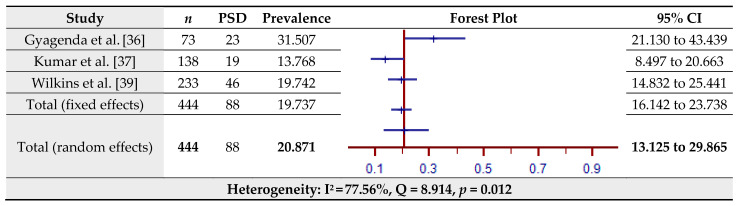
Prevalence estimates of Post-Stroke Depression (PSD) assessed by the Patient Health Questionnaire (PHQ-9).

The estimated prevalence of PSD assessed by the PHQ-9 for four studies is shown in Figure 4. The pooled prevalence of PSD in the total sample of 544 was 45.55% (95% CI = 6.29–88.71%) based on the random-effects model (I^2^ = 99.21%, Q = 379.61, *p* < 0.001). Since Litton et al. [38] provided us with a 100% prevalence, thereby appearing as an outlier, the prevalence of PSD as assessed by the PHQ-9 including the aforementioned study can be found separately in Figure 4. The estimated prevalence of PSD assessed by the PHQ-9 for three studies (without Litton et al. [38]) is shown in Figure 5. The pooled prevalence of PSD in the total sample of 444 was 20.87% (95% CI = 13.13–29.87%) based on the random-effects model (I^2^ = 77.56%, Q = 8.91, *p* = 0.012).

### 3.5. Prevalence of Post-Stroke Depression (PSD) Assessed by the Schedules for Clinical Assessment in Neuropsychiatry (SCAN)

**Figure 6:**
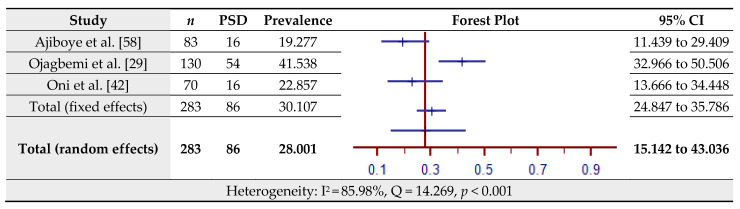
Prevalence estimates of Post-Stroke Depression (PSD) assessed by the Schedules for Clinical Assessment in Neuropsychiatry (SCAN).

The estimated prevalence of PSD assessed by the SCAN for three studies is shown in Figure 6. The pooled prevalence of PSD in the total sample of 283 was 28.00% (95% CI = 15.14–43.04%) based on the random-effects model (I^2^ = 85.98%, Q = 14.27, *p* < 0.001).

### 3.6. Prevalence of Post-Stroke Depression (PSD) Assessed by the Geriatric Depression Scale (GDS-15/30)

**Figure 7:**
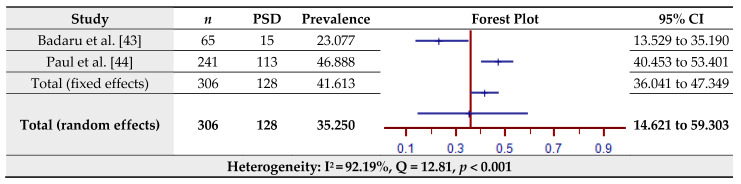
Prevalence estimates of Post-Stroke Depression (PSD) assessed by the Geriatric Depression Scale (GDS15/30).

The estimated prevalence of PSD assessed by the GDS-15/30 for two studies is shown in Figure 7. The pooled prevalence of PSD in the total sample of 306 was 35.25% (95% CI = 14.62–59.30%) based on the random-effects model (I^2^ = 92.19%, Q = 12.81, *p* < 0.001).

### 3.7. Prevalence of Post-Stroke Depression (PSD) Assessed by the Montgomery–Asberg Depression Rating Scale (MADS)

**Figure 8:**
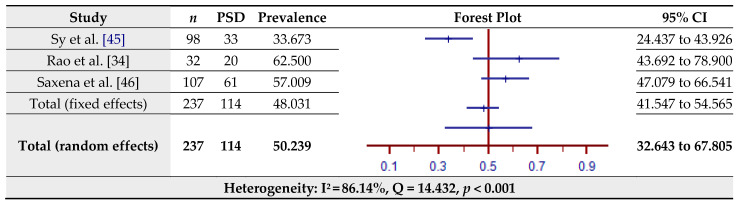
Prevalence estimates of post-stroke depression (PSD) assessed by the Montgomery–Asberg Depression Rating Scale (MADS).

The estimated prevalence of PSD assessed by the MADS for three studies is shown in Figure 8. The pooled prevalence of PSD in the total sample of 237 was 50.24% (95% CI = 32.64–67.81%) based on the random-effects model (I^2^ = 86.14%, Q = 14.43, *p* < 0.001).

### 3.8. Prevalence of Post-Stroke Anxiety (PSA) Assessed by the Hospital Anxiety and Depression Scale (HADS)

**Figure 9:**
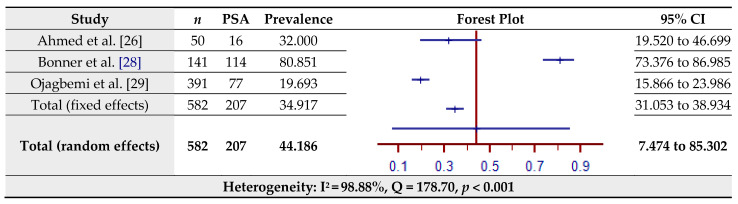
Prevalence estimates of Post-Stroke Anxiety (PSA) assessed by the Hospital Anxiety and Depression Scale (HADS).

The estimated prevalence of PSA assessed by the HADS for three studies is shown in Figure 9. The pooled prevalence of PSA in the total sample of 582 was 44.19% (95% CI = 7.47–85.30%) based on the random-effects model (I^2^ = 98.88%, Q = 178.70, *p* < 0.001).

### 3.9. Prevalence of Post-Stroke Anxiety (PSA) Assessed by the Schedules for Clinical Assessment in Neuropsychiatry (SCAN)

**Figure 10:**
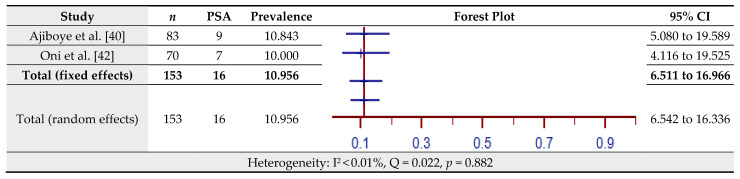
Prevalence estimates of Post-Stroke Anxiety (PSA) assessed by the Schedules for Clinical Assessment in Neuropsychiatry (SCAN).

The estimated prevalence of PSA assessed by the SCAN for two studies is shown in Figure 10. The pooled prevalence of PSA in the total sample of 153 was 10.96% (95% CI = 6.511–16.97%) based on the fixed-effects model (I^2^ < 0.01%, Q = 0.22, *p* = 0.882).

### 3.10. Prevalence of Post-Stroke (PS) Cognitive Decline Assessed by the Mini-Mental Status Examination (MMSE)

**Figure 11:**
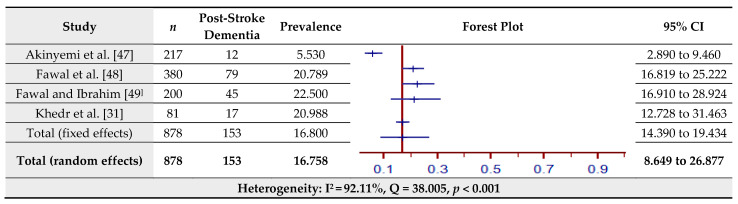
Prevalence estimates of post-stroke cognitive decline assessed by the Mini-Mental Status Examination (MMSE).

The estimated prevalence of PS Dementia assessed by the MMSE for four studies is shown in Figure 11. The pooled prevalence of PS Dementia in the total sample of 878 was 16.76% (95% CI = 8.65–26.88%) based on the random-effects model (I^2^ = 92.11%, Q = 38.01, *p* < 0.001).

### 3.11. Exclusion of Certain Studies

The current meta-analysis was done using a subgroup analysis based on the tool used for the assessment of prevalence. It is important to note that, while most of the studies tap only into subthreshold symptoms or probable ‘case-ness’ for both psychiatric and cognitive symptoms, there are some exceptions. In the current review, three studies utilized the Schedules for Clinical Assessment in Neuropsychiatry (SCAN) to extrapolate post-stroke data [29,40,42]. The SCAN has been designed to diagnose multiple psychiatric conditions among adults. Since measured conditions other than post-stroke anxiety and depression could not be grouped with other studies utilizing the same tool, they were not considered for this review. 

Moreover, it was also not possible to group certain high-quality studies into any of the tool-based categories, since each study covered a single unique disorder by itself (i.e., post-stroke delirium, sleep disorders, and aphasia). Additionally, studies were also excluded even if they did reliably measure any of the disorders included in the current meta-analysis but utilized a unique tool that was not used by at least one other study. 

## 4. Discussion

While the industrialized countries of North America and Western Europe have mobilized multiple strategies for the prevention of cardiovascular diseases and the mitigation of its sequelae, i.e., post-stroke disability and quality of life, the trend appears, in general, to be reversed in the current countries under scrutiny [65]. The presently defined region of interest included several countries comprising West Asia, South Asia, and Africa. A majority of these countries have been widely documented to have a population characterized by the commonly known risk factors preceding a stroke incident [6,12]. In this regard, the prevalence of stroke survivors has also been increasing globally with prevalence edging on the higher side in countries of the aforementioned region. As per a report by the World Health Organization (WHO) i.e., *Rehabilitation 2030: A call for action* [9], the WHO has recently embarked on a task to ‘upscale’ rehabilitation globally to address such unmet needs in developing countries [66]. Within the background of the anticipated ‘rehaul’ of stroke rehabilitation in developing countries, this critical appraisal examined the prevalence of psychiatric and cognitive symptoms among stroke survivors. It has been estimated that by 2030, approximately 70 million people across the world will be living with an intransigent and debilitating sequela of stroke that broadly falls into physical, psychiatric, and cognitive domains. While the concepts of post-stroke psychiatric and cognitive symptoms are sometimes amorphous compared to physical disability, the mere presence of such symptoms has been widely established to impede recovery by reducing stroke survivor adherence to the rehabilitation process and the required lifestyle regimen. 

The articles that fulfill the present inclusion criteria were those from West Asia (Saudi Arabia, Qatar, Jordan, Iran), the Indian subcontinent (India, Sri Lanka, and Bangladesh), and Africa (Egypt, Nigeria, Senegal, Uganda). A majority of these articles were observational studies that reported mental disorders and cognitive impairment and employed common questionnaires such as the Hospital Anxiety and Depressive Scale (HADS), Hamilton Depression Rating Scale (HDRS), Patient Health Questionnaire (PHQ-9), Geriatric Depression Scale (GDS15/30), and the Montgomery–Asberg Depression Rating Scale (MADS). These questionnaires, which are sometimes known to be administered as a part of structured interviews, are known to reveal probable diagnosis or subthreshold psychiatric symptoms. However, three studies utilized the more robust semi-structured interview process (Schedules for Clinical Assessment in Neuropsychiatry, SCAN). Some studies tapping into cognitive symptoms did not employ conventional neuropsychological batteries but rather used ‘bedside measures’ such as the Mini-Mental Status Examination (MMSE), which are only equipped to indicate the presence of cognitive decline. 

In this study, post-stroke duration referred to the amount of time following the stroke incident after which the participants of each study were investigated for secondary conditions. In the three accrued articles, it was found that post-stroke duration was extremely varied, ranging from 1 week up to 10 years [33,46]. In this regard, it is essential to note that there are currently no established guidelines stating when it is optimal to conduct psychiatric and cognitive assessment following a cardiovascular accident. Robinson and Jorge [11] have stated that psychiatric and cognitive assessments tend to be employed “a few weeks following stroke to 6 or more months following stroke” (p. 224). Relevant to the time since injury and initiation of testing, it is a noteworthy fact that psychiatric and cognitive symptoms are not static. A majority of stroke survivors tend to witness spontaneous recovery throughout their lives, while some others plateau. Stroke may also trigger irreversible neurodegenerative processes among certain survivors [67]. Therefore, there is a need to make a clear demarcation of what constitutes an acute and chronic phase post-stroke. The ensuing paragraphs examine the pooled prevalence of post-stroke depression, post-stroke anxiety and post-stroke cognitive impairment in context of the instruments used.

### 4.1. Post-Stroke Depression (PSD)

It has been widely recognized that the presence of depressive symptoms in post-stroke survivors tends to adversely affect the road to recovery, attenuating self-directedness and quality of life and heightening health service utilization and the risk of early death [41,68,69,70,71]. There are several systematic reviews and meta-analyses on PSD. Ayerbe, Ayis, Wolfe and Rudd [15] surveyed studies up to August 2011, identifying 50 articles (*n* = 20293) and yielding a pooled prevalence of 29%. In their systematic review that included 61 prospective observational studies up to May 2013 (*n* = 25,488), Hackett and Pickles [16] reported a pooled prevalence rate of 31%. In the present review, the depression subscale of HADS was used in four studies for detecting post-stroke depressive tendencies. The pooled frequency of PSD was 46.17% (*n* = 780). Seven studies (*n* = 658) utilized the Hamilton Depression Rating Scale (HDRS), yielding a pooled prevalence of 50.09%. With a pooled prevalence rate of 45.55%, four studies (*n* = 544) used the Patient Health Questionnaire (PHQ-9). The Geriatric Depression Scale (GDS-15/30) was used to assess depressive symptoms post-stroke in two studies (*n* = 306), giving a pooled prevalence of 35.25%. Three other studies (*n* = 237) used the Montgomery–Asberg Depression Rating Scale (MADS), yielding a pooled prevalence rate of 50.24%. Finally, only three remaining studies utilized semi-structured interviews, or Schedules for Clinical Assessment in Neuropsychiatry (SCAN) (*n* = 263), to tap into PSD. The pooled prevalence rate was 30.43%. It appears that the utilization of questionnaires tends to give spurious results compared to the semi-structured interview, often viewed as the gold standard for diagnosing depression. Overall, the present region of interest suggests that the rate of depressive symptoms ranged from 30.43 to 50.24%, which appears to be in the same range as that reported in previous meta-analyses, i.e., 29–31% [15,16]. Previous studies have alluded to the view that depressive symptoms in the non-Western population, due to cultural patterning, manifest differently from the Western population [72]. The present data appears to challenge such an assumption, since many of the instruments used in the current studies, except for the HADS, were those that predominantly tap into emotional symptoms [73]. 

### 4.2. Post-Stroke Anxiety (PSA)

One hypothesis is that anxiety constitutes a risk for stroke by approximately 24% [74], but there is also a dissenting view that stroke is precipitated by episodic paroxysmal anxiety [75]. However, the occurrence of post-stroke anxiety disorders has been widely documented. The presence of anxiety in stroke survivors shows poor prognostic indicators including the reduced quality of life and recurrence of stroke and death [76,77]. Anxiety disorders also augment the adverse effects of depressive symptoms [78]. Burton et al. [79] conducted a systematic review and meta-analysis of observational studies up to March 2011. Forty-four studies (*n* = 5760) with stroke survivors fulfilled the study criteria. The pooled estimate of anxiety disorders stood at 18%. Rafsten, Danielsson, and Sunnerhagen [80] conducted a systematic review and meta-analysis for the first year after stroke, covering studies up to April 2017 (*n* = 13,756). The pooled prevalence of anxiety disorders was 29.3%. Knapp et al. [81] have reported the results of a systematic review and meta-analysis of 97 observational studies (*n* = 22,262) of anxiety after stroke. Two types of the pooled estimates emerged: the semi-structured interview revealed 18.7% to be marked with anxiety disorder while the use of a symptom checklist yielded a prevalence of 24.2%. In the present review, both symptom checklists (HADS) and semi-structured interviews (SCAN) were used. A pooled prevalence of 44.19% for HADS was calculated from three studies (*n* = 582). Given that semi-structured interviews are more robust in identifying anxiety disorders, as often articulated in the literature, the pooled estimate derived from the two studies included in the present review (*n* = 153) was only 10.96%.

### 4.3. Post Stroke Cognitive Impairment (PSCI)

Cognitive impairment, characterized by impaired attention and concentration, problems with learning and remembering, executive functioning, the efficiency of speech and language and visual–spatial ability [21], has been placed under a new diagnostic category—‘major cognitive disorders’—in the Diagnostic and Statistical Manual of Mental Disorders-V (DSM-5). Cognitive impairment is often accompanied by overt structural and functional changes in the brain among stroke survivors [82] and thus constitutes an organic, as opposed to a functional, disorder [83]. Post-stroke cognitive impairment is also accompanied by weakened functionality which, in turn, has a direct bearing on quality of life and the sense of a meaningful existence [84]. Post-stroke cognitive impairment often culminates in major cognitive disorders, formerly known as dementia [85]. The prevalence of PSCI has been estimated to vary from 22 to 47% [86,87]. Subtle yet intransigent and deliberating, PSCI has received scant attention in the existing literature. A major reason for this is that normative data for conventional neuropsychological batteries are yet to be established in a majority of the countries in West and South Asia and Africa [88]. It is, therefore, no surprise that the majority of the evaluations of PSCI were done using quick and easy measures of cognitive decline such as the MMSE. The present review accrued four studies (*n* = 878) with a pooled estimate of 16.76%. This rate appears to be in the lower range of the global trend ranging from 22 to 47%.

### 4.4. Limitations

In addition to the limitations often associated with systematic reviews and meta-analyses, the studies that have emerged from West and South Asia and Africa on post-stroke psychiatric and cognitive symptoms have various limitations of their own that need to be highlighted. Such limitations could be used as part of the background to strengthen the approach to the field in these aforementioned regions. Firstly, in the literature, there has been a widespread interest in cognition and psychiatry-specific instruments. It is not clear whether the diverse instruments employed in the present region of interest have been subjected to evaluation based on local psychometric properties. Relevant to this in the context of cognitive functioning, it appears that the MMSE has been widely used in the present region of interest. However, there are indications that this instrument is fairly weak in terms of sensitivity to minor cognitive impairment among the post-stroke population [89,90]. This should certainly be considered as one of the limitations of the studies that utilized this measure included in the current meta-analysis. 

Secondly, in some of the articles considered for the present review, there were many instances where it was not explicitly clear whether the quantification of post-stroke symptoms was done via self-reported instruments or through other means. Related to this, it was also not apparent whether the clinician-based interviews were conducted under the guidance of the established gold-standard interview protocol or simply under the clinical impression of the researcher/clinicians. Some of the assessment measures such as the Hamilton Depression Rating Scale have been widely reported as being part of the semi-structured interview process in a clinician-administered depression assessment scale, but there is a dissenting view in the literature [91]. Future studies from developing countries must elucidate whether the targeted symptoms were gauged via a semi-structured interview, a clinician-administered assessment scale, or self-reported questionnaires. This would lay the groundwork for establishing the evidence-based prevalence of post-stroke psychiatric symptoms.

Future studies should also examine the feasibility of developing disease-specific instruments to identify post-stroke psychiatric and cognitive complications. Societies belonging to the presently defined region are diverse in terms of ethnicity, language, and culture. It is not clear whether the evaluation of post-stroke psychiatric and cognitive symptoms was performed while keeping the local idioms of distress in mind, since many of them were developed in industrialized countries. Third, it is likely that some of the studies that emerged from these vastly diverse regions have been published in languages other than English. This critical appraisal has overlooked such articles, and this should certainly be considered an important confounder. Fourth, it is commendable that research from the presently considered regions has been conducted using proper strategies for documenting post-stroke psychiatric and cognitive symptoms given the circumstances. However, it was noted that the quality and quantity of these research papers needed to be improved and increased. For instance, concerted efforts should be made to chart out premorbid functioning, pre-existing cognitive and psychiatric symptoms, single or multiple strokes, subtypes of strokes, as well as issues pertinent to localization and lateralization. It may also be worthwhile to explore the relationship between motor/functional conditions and psychiatric and cognitive conditions secondary to stroke. Fifth, while it has been widely suggested that post-stroke survivors tend to manifest various psychiatric and neuropsychiatric symptoms, in the present region of interest, most of the research focused predominantly on symptoms of anxiety and depression. Further studies quantifying other types of neurobehavioral impairments (e.g., apathy, disinhibition, executive dysfunction) are warranted [92,93].

## 5. Conclusions

The present region of interest that is often alternatively labelled the ‘global south’ or simply ‘developing countries’ has been widely described as being plagued with two health threats. One of them includes the traditional enemies of health such as malnutrition, child mortality, and environment-related and infectious diseases, while the other includes non-communicable diseases that entail, for example, neurological events that lead to the debilitating sequelae of focal (or global) disturbance in cerebral function. Around 80% of the global population resides in these regions and while existing rudimental emergency services and critical care often lead to high death rates among the majority of those sustaining stroke, a significant number of them have gone on to survive, though with severely debilitating conditions. The present review has suggested that both psychiatric and cognitive symptoms are common among stroke survivors in the regions of West and South Asia and Africa, and form part of the unmet needs of such survivors. Concerted efforts are needed to institute more robust studies using culturally sensitive measures to contemplate mechanisms that would address the needs of these vulnerable populations.

## Figures and Tables

**Table 1:** Evaluation of the Qualifying Papers using the JBI Guidelines.

Study	Q1 ^1^	Q2 ^2^	Q3 ^3^	Q4 ^4^	Q5 ^5^	Q6 ^6^	Q7 ^7^	Q8 ^8^
Ahmed et al. (2020) [26]	Y	Y	Y	Y	Y	Y	Y	Y
Ayasrah et al. (2018) [27]	Y	Y	Y	Y	Y	Y	Y	Y
Bonner et al. (2016) [28]	Y	Y	N	Y	Y	N	Y	Y
Ojagbemi et al. (2017) [29]	Y	Y	Y	Y	Y	Y	Y	Y
Islam et al. (2016) [30]	Y	Y	N	Y	Y	N	Y	Y
Khedr et al. (2020) [31]	Y	Y	Y	Y	Y	U	Y	Y
Pasha et al. (2019) [32]	Y	Y	Y	Y	Y	N	Y	Y
Patel et al. (2018) [33]	Y	Y	Y	Y	Y	N	Y	Y
Rao et al. (2014) [34]	Y	Y	U	Y	Y	N	Y	Y
Srivastava et al. (2010) [35]	Y	Y	Y	Y	Y	N	Y	Y
Gyagenda et al. (2015) [36]	Y	Y	Y	Y	Y	N	Y	Y
Kumar et al. (2020) [37]	Y	Y	Y	Y	Y	N	Y	Y
Litton et al. (2016) [38]	Y	N	Y	Y	Y	N	Y	Y
Wilkins et al. (2018) [39]	U	Y	Y	Y	Y	Y	Y	Y
Ajiboye et al. (2013) [40]	Y	Y	Y	Y	Y	Y	Y	Y
Ojagbemi et al. (2019) [41]	Y	Y	Y	Y	Y	Y	Y	Y
Oni et al. (2018) [42]	Y	Y	Y	Y	N	N	Y	Y
Badaru et al. (2013) [43]	Y	N	N	Y	Y	Y	Y	Y
Paul et al. (2013) [44]	Y	Y	Y	Y	Y	N	Y	Y
Sy et al. (2019) [45]	Y	Y	Y	Y	N	N	Y	Y
Saxena et al. (2015) [46]	Y	Y	Y	Y	Y	N	Y	Y
Akinyemi et al. (2014) [47]	U	Y	Y	Y	Y	U	Y	Y
Fawal et al. (2021) [48]	Y	Y	Y	Y	Y	Y	Y	Y
Fawal and Ibrahim (2015) [49]	Y	Y	Y	Y	N	N	Y	Y
Khedr et al. (2009) [50]	Y	Y	Y	Y	Y	N	Y	Y

^1^ Were the criteria for inclusion in the sample clearly defined? ^2^ Were the study subjects and the setting described in detail? ^3^ Was the exposure measured in a valid and reliable way? ^4^ Were objective, standard criteria used for the measurement of the condition? ^5^ Were confounding factors identified? ^6^ Were strategies to deal with confounding factors stated? ^7^ Were the outcomes measured in a valid and reliable way? ^8^ Was appropriate statistical analysis used?

**Table 2:** Characteristics of the studies included for systematic review.

#	Author (Year)	Country	Year of Study	Study Design	Sampling Method	Age (Years) [Mean (SD)]	Setting	Sample Size	Gender (M/F)	Screening Tool	Disorder Screened	Positive Cases	Post-Stroke Duration
1	Ahmed et al. (2020) [26]	Saudi Arabia	2020	Cross-sectional	Convenience Sampling	56.72 (11.83)	Hospital	50	28/22	HADS	PSA PSD	PSD = 18 PSA = 16	3 Months
2	Wilkins et al. (2018) [39]	Qatar	2016–2017	Cross-sectional	Convenience Sampling	PHQ-9 Positive: 52.56 (10.37) PHQ-9 Negative: 49.81 (11.32) Median = NA Range = NA	Hospital	233	202/31	PHQ-9	PSD	PSD = 46	First Few Days After Stroke
3	Ayasrah et al. (2018) [27]	Jordan	2017	Cross-sectional	Convenience Sampling	56.62 (14.2)	Hospital	198	110/88	HADS-D	PSD	PSD = 151	Upto 3 Months
4	Rezaei et al. (2016) [54]	Iran	2012–2014	Retrospective cohort	Convenience Sampling	PSCI: 74.52 (8.8) No PSCI: 61.49 (10.78)	Hospital	206	14/34	SCID	PSCI	PSCI = 48	2 Years
5	Mansour et al. (2020) [55]	Egypt	2015	Cross-sectional	Convenience Sampling	59.3 (5.34)	Hospital	75	34/41	PSQI; ESS; AHI	PS Sleep Disorder	PS Sleep Disorders = 66	3 Months
6	Khedr et al. (2020) [31]	Egypt	2014–2015	Cross-sectional	Convenience Sampling	Patients: 61.2 (14.7) Controls:59 (13.3)	Hospital (Controls: 2nd-degree relatives)	153	Patients: 62/41 Controls: 32/18	SCID-CV; HDRS	PSD	PSD (Patients): 38/103 PSD(Controls):6/50	Onset–2 years
7	Khedr et al. (2009) [50]	Egypt	Unavailable	Longitudinal	Convenience Sampling	57.7 (5.19)	Hospital	81	54/27	MMSE; CASI; WM-R; DSM-IV	PSCI PSD	PSCI = 17 PSD = 20	3 Months
8	Fawal, Ibrahim and Elhamed (2021) [48]	Egypt	2017–2019	Prospective cohort	Convenience Sampling	PSCI: 71.63 (9.8) No PSCI: 58.90 (11.6)	Hospital	380	PSCI: 42/37 No PSCI: 170/131	MMSE; DSM 5	PSCI	PSCI = 79	6 Months
9	Fawal and Ibrahim (2015) [49]	Egypt	2014	Cross-sectional	Convenience Sampling	Male: 62.5 (NA) Female:59.2 (NA)	Hospital	200	112/88	MMSE, HDRS	PSCI PSD;	PSCI = 45 PSD = 56	3 Months
10	Ojagbemi et al. (2019) [41]	Nigeria	2010	Cross-sectional	Convenience Sampling	59.8 (11)	Hospital	Patients: 130 Controls: 130	Patients: 60/70 Controls: 63/67	SCAN, MMSE, MIUTT	PSD	PSD = 54 Global cognitive dysfunction = 34 Executive dysfunction = 101	3 Months–2 Years
11	Oni et al. (2018) [42]	Nigeria	2013–2014	Case-control cross-sectional study	Convenience Sampling	Patients: 57.43 (9.67) Controls: 57.33 (9.33)	Hospital	Patients:70 Controls: 70	Patients: 38/32 Controls: 38/32	MMSE, SCAN, mRS, WHOQoLBREF	PSD	PSD = 16	<1–>2 Years
12	Oni et al. (2017) [56]	Nigeria	2013–2014	Cross-sectional	Convenience Sampling	57.43 (9.67)	Hospital	70	38/32	SCAN	PSA	PSAD = 7	<1–≥ 1 Year
13	Abubakar et al. (2012) [57]	Nigeria	2009–2010	Cross-sectional and Descriptive Correlational Design	Convenience Sampling	54.4 (9.9)	Hospital	62	33/29	ZDS	PSD	PSD = 18	3 Months
14	Ajiboye et al. (2013) [40]	Nigeria	2009–2010	Cross-sectional	Convenience Sampling	60.6 (13.2)	Hospital	83	37/46	SCAN and ICD-10	PSD PSA	PSD = 16 PSA = 9	<1 Year– >10 Years
15	Ojagbemi et al. (2017) [29]	Nigeria	NA	Cross-sectional (sample drawn from RCT)	Convenience Sampling	57.3 (11.7)	Hospital	391	249/142	HADS-A	PSA	PSA = 77	Within1 Month
16	Fatoye et al. (2009) [58]	Nigeria	NA	Cross-sectional	Convenience Sampling	Patients: 59.6 (10.5) Controls: 58.4 (10.2)	Hospital (Controls: Hypertensive patients)	236	137/99	BDI	PSD	PSD = 47	1 Month–2 Years
17	Olibamoyo et al. (2019) [59]	Nigeria	NA	Cross-sectional	Simple Random Sampling	56.71 (6.49)	Hospital	112	67/45	MINI	PSD	PSD = 48	6 Months
18	Imarhiagbe et al. (2015) [60]	Nigeria	NA	Cohort Study	Convenience Sampling	63.79 (13.36)	Hospital	92	61/31	JSS–D; DSM-IV	PSD	PSD = 17	10 Months (median)
19	Ojagbemi et al. (2017) [61]	Nigeria	NA	Longitudinal Observation Study	Convenience Sampling	Baseline: 61.1 (12.9)	Hospital	99	52/47	CAM;DRS	PS Delirium	PS Delirium = 33	3 Months
20	Akinyemi et al. (2014) [47]	Nigeria	2010–2012	Comprehensive Prospective Study	Convenience Sampling	60.4 (9.5)	Hospital	217	0/ 89	CSID; MMSE; V-NB	PSCI	PSCI = 12	3 Months
21	Badaru et al. (2013) [43]	Nigeria	NA	Cross-sectional Survey	Purposive Sampling	Range = 58–80	Hospital	65	37/28	GDS-15 (Short Version)	PSD	PSD = 15	1–4 Years
22	Sy et al. (2019) [45]	Senegal	2016	Prospective Cross-sectional Study	Convenience Sampling	58.5 (13.7)	Hospital	98	55/43	DSM-V MADRS	PSD	PSD = 33	3–6 Months
23	Gyagenda et al. (2015) [36]	Uganda	2014	Cross-sectional Study	Convenience Sampling	Range = 20–99	Hospital	73	30/43	PHQ–9; ADRS	PSD	PSD = 23	3–12 Months
24	Saxena et al. (2015) [46]	India	2013	Cross-sectional Study	Convenience Sampling	59.13 (11.66)	Hospital	107	60/47	MADRS	PSD	PSD = 61	1 Week
25	Srivastava et al. (2010) [35]	India	2010	Cross-sectional	Convenience Sampling	46.06 (11.19)	Hospital	51	41/10	HRDS	PSD	PSD = 18	>3 Months
26	Patel et al. (2018) [33]	India	2015–2016	Cross-sectional	Convenience Sampling	Range = 20 and above	Hospital	52	38/14	HAM-D	PSD	PSD = 38	1 Month–10 Years
27	Paul et al. (2013) [44]	India	2006–2010	Prospective cohort study	Stratified random Sampling	62.7 (13.04)	Community-based stroke registry	241	118/123	bGDS	PSD	PSD = 113	3–18 Months
28	Kumar et al. (2020) [37]	India	NA	Cross-sectional	Consecutive Sampling	Range = 18–70	Hospital	138	91/47	PHQ-9	PSD	PSD =19	1 Month
29	Litton et al. (2016) [38]	India	2014–2015	Cross sectional observational	convenience Sampling	63.84 (11.40)	Hospital	100	68/32	PHQ-9	PSD	PSD = 100	2 Weeks
30	Rao et al. (2014) [34]	India	NA	Prospective	Consecutive Sampling	46.25 (NA)	Hospital	32	19/13	HAM-D; MADRS	PSD	PSD: 2 weeks = 20 1 month = 19 3 month = 13	2 Weeks–3 Months
31	Pasha et al. (2018) [32]	India	NA	Cross-sectional	Convenience Sampling	NA	Hospital	56	38/18	DSM-IV TR; HAM-D	PSD	PSD = 26	3–12 Months
32	Bonner et al. (2016) [28]	India	2012–2013	Surveys	Consecutive Sampling	48 (8.8)	Hospital	141	138/3	HADS	PSA PSD	PSD = 86 PSA = 114	3 Months–2 Years
33	Gupta et al. (2014) [62]	India	2011–2012	Prospective, observational	Convenience Sampling	58.8 (12.03)	Hospital	60	40/20	NPI; CDR	NI	NI = 57	At Least 3 Months
34	Lahiri et al. (2020) [63]	India	2016–2018	Observational study	Consecutive	52.19 (10.96)	Hospital	515	299/216	BWAB	PS Aphasia	PS Aphasia= 208	7–14 Days
35	Isuru et al. (2021) [64]	Sri Lanka	2014–2017	Cross-sectional descriptive	Convenience Sampling	Range: <30 years–>70 years	Hospital	374	155/219	ICD-10	PSD	PSD = 106	Within 1 Month
36	Islam et al. (2016) [30]	Bangladesh	2011	Cross-sectional	Consecutive Sampling	58.91 (7.03)	Hospital	164	112/52	HAM-D	PSD	PSD = 115	1–3 Months and >3 Months

HADS = Hospital Anxiety Depression Scale; PS = Post-stroke; PSD = Post-Stroke Depression; PSA = Post-Stroke Anxiety; PSCI = Post-Stroke Cognitive Impairment; mRS = Modified Rankin Scale; PHQ-9 = Patient Health Questionnaire; SCID = Structured Clinical Interview (in accordance with DSM-5); SCID-CV = Structured Clinical Interview for DSM-IV-Clinician Version; PSQI = Pittsburg Sleep Quality Index; ESS = Epworth sleepiness scale; AHI = Apnea/Hypopnea Index; HDRS = Hamilton Depression Rating Scale; WHOQOL-BREF = World Health Organization Quality of Life; BI = Barthel Index; MMSE = Mini-Mental State Examination; CASI = Cognitive Abilities Screening Instruments; WMS-R = Wechsler Memory Scale-Revised; SCAN = Schedules for Clinical Assessment in Neuropsychiatry; MMSE = Mini Mental Status Examination; HAM-D = Hamilton Rating Scale for Depression; bGDS = Bengali version of the Geriatric Depression Scale; MADRS = Montgomery–Asberg Depression Rating Scale; DSM-IV-TR = Developing the Structured Clinical Interview; NPI = Neuropsychiatric Inventory; CDR = Clinical Dementia Rating Scale; BWAB = The Bengali version of Western Aphasia Battery; ICD-10 = International Statistical Classification of Diseases; CSID = Community Screening Instrument for Dementia; GDS-15 = Geriatric Depression Scale-15; ADRS = Aphasic Depression Rating Scale; BIADL = Barthel Index of Activities of Daily Living; ZDS = Zung Self-Rating Depression Scale; BDI = Beck’s Depression Inventory; JSS-D = Japanese Stroke Scale for Depression; CAM = Confusion Assessment Method; DRS = Delirium Rating Scale; MINI = Mini International Neuropsychiatric Interview; NI = Neurobehavioral Impairment; MIUTT = modified Indiana University Token test.

## Data Availability

All data generated and analysed during this study are included as part of this article.
